# How do biting disease vectors behaviourally respond to host availability?

**DOI:** 10.1186/s13071-016-1762-4

**Published:** 2016-08-25

**Authors:** Laith Yakob

**Affiliations:** Department of Disease Control, Faculty of Infectious and Tropical Diseases, London School of Hygiene and Tropical Medicine, Keppel Street, London, WC1E 7HT UK

**Keywords:** Behaviour ecology, Functional response, Vector-borne disease, Malaria, Chagas disease, Lyme disease

## Abstract

**Background:**

Ecological theory predicts a diverse range of functional responses of species to resource availability; but in the context of human blood consumption by disease vectors, a simplistic, linear response is ubiquitously assumed. A simple and flexible model formulation is presented that extends the Holling’s Types to account for a wider range of qualitatively distinct behaviours, and used to examine the impact of different vector responses to the relative availability of multiple blood-host species.

**Results:**

Epidemiological models of falciparum malaria, Chagas disease and Lyme disease demonstrate that the standard, often implicit, assumption of a linear functional response can lead to spurious under- or over-estimates in disease transmission potential, across a full range of pathogen life-cycles. It is shown how the functional response in vector biting can augment disease intervention outcomes. Interactions between vector biting behaviour and uneven pathogen transmission probabilities between alternative hosts, as is the case for Chagas disease, can render infection more resilient to control.

**Conclusions:**

Both the novel response formula and the nested vector-borne disease structure offer a flexible framework that can be applied to other vector-borne diseases in assessing the role of this newly identified aspect of biting behavioural ecology.

## Background

How species respond to availability in resources is highly variable and has fostered considerable interest among ecologists for decades. Seminal papers written by Holling describe the different forms of functional response that predators exhibit to prey density [1–3]. A term first coined by Solomon [4], ‘functional response’ refers to the influence of resource availability on the rate of its consumption. Three qualitatively distinct functional responses were originally described: Type I responses depict resource consumption as a linear function of availability; Type II responses depict resource consumption as a decelerating function of availability (convex-up); and Type III responses depict resource consumption as an initially accelerating but then decelerating function of availability (s-shaped curve). Although Holling initially drew strong delineation between the types of predator species and the functional responses that they exhibit (e.g. invertebrates are Type II whereas mammals are Type III), subsequent ecological studies have generalised this phenomenon beyond predator-prey interactions (to account for all manner of resource consumption) and recast various species across a continuous spectrum of Types [5].

Real [6] devised a general formula that enabled flexible characterisation of a Hollings Type I, II or III response. This constituted an important contribution because different response Types could be assessed simply through re-parameterisation of the same underlying model, and this type of nesting is an advantage in direct comparison between models in estimating best fit to data [7]. The formulation proposed by Real [6] is as follows:

1 $$ C=\frac{\alpha {N}^{\beta }}{1+\alpha w{N}^{\beta }} $$

where, *C* is the resource consumption rate, *N* is the density (or availability) of the resource, *w* is the handling time of the resource (i.e. the amount of time taken between identifying the prey and consuming it), and α and β are shape parameters. In the case that *w* = 0 and β = 1, *C* = α*N* (a linear Type I response); in the case that *w* > 0 and β = 1, a formulation which is equivalent to the Michaelis-Menten equation of enzyme kinetics results and is characterised by a decelerating consumption that eventually saturates (Type II); in the case that *w* > 0 and β > 1, a sigmoidal relationship results (Type III).

However, the extent of analogous developments in the context of vector-borne diseases is very limited. Many disease vectors are obligatorily haematophagous, meaning blood is a critical resource for survival and/or reproduction [8]. An important distinction to make here is that host death would rarely be expected as a direct consequence of blood-feeding by a vector (an exception being neurotoxin-mediated tick paralysis), but instead may result from coincident pathogen transmission. Thus the bidirectional effects on both consumer and resource species population dynamics implicit to predator/prey (and parasitoid/host) systems do not necessarily hold here. A further necessary consideration for systems of haematophagous disease vectors is that, as a rule, blood can be (and is) sourced from multiple host species. Even the most discerning of vector species thrive on the blood of multiple hosts [9, 10]. For example, *Anopheles gambiae* (*sensu stricto*) famously shows extreme preference for human blood [11] but adapts to environments with low human availability by sourcing blood from alternative mammals [12] with reportedly little-to-no effect on its resulting fecundity [13]. This is in stark contrast to parasitoid systems which are typically highly specialised and to most predator-prey theoretical and empirical studies (although, see multi-species models of Abrams & Matsuda [14] and Rueffler et al. [15]). To avoid conflation, the biting response of disease vectors to host availability shall be referred to as ‘behavioural’ instead of ‘functional’.

Development in the understanding of behavioural responses in disease vector biting is timely as this field is anticipated to accelerate rapidly following recent advances in molecular approaches; novel biological fingerprinting methods allow for the inexpensive, rapid and sensitive identification of host species from vector blood meals [16]. In the context of human disease control, there has been a recently rekindled interest in targeting vectors that bite alternative (often domestic) host species [17–19]. However, the full potential of these advances in informing vector-borne disease epidemiology will only be realised with their appropriate interpretation through a more developed ecological theory. Of the few ecological and epidemiological studies that consider host preference among vectors, linearity between alternative host availability and vector response is typically assumed [17, 20, 21]. In other words, a doubling in the availability of a particular host species relative to all potential hosts doubles the proportion of bites taken on that species. Here, the behavioural response of haematophagous arthropod disease vectors to the availability of alternative hosts and the resulting consequences for vector-borne disease transmission are explored.

## Methods

### Modelling the behavioural response in biting disease vectors

**Fig. 1:**
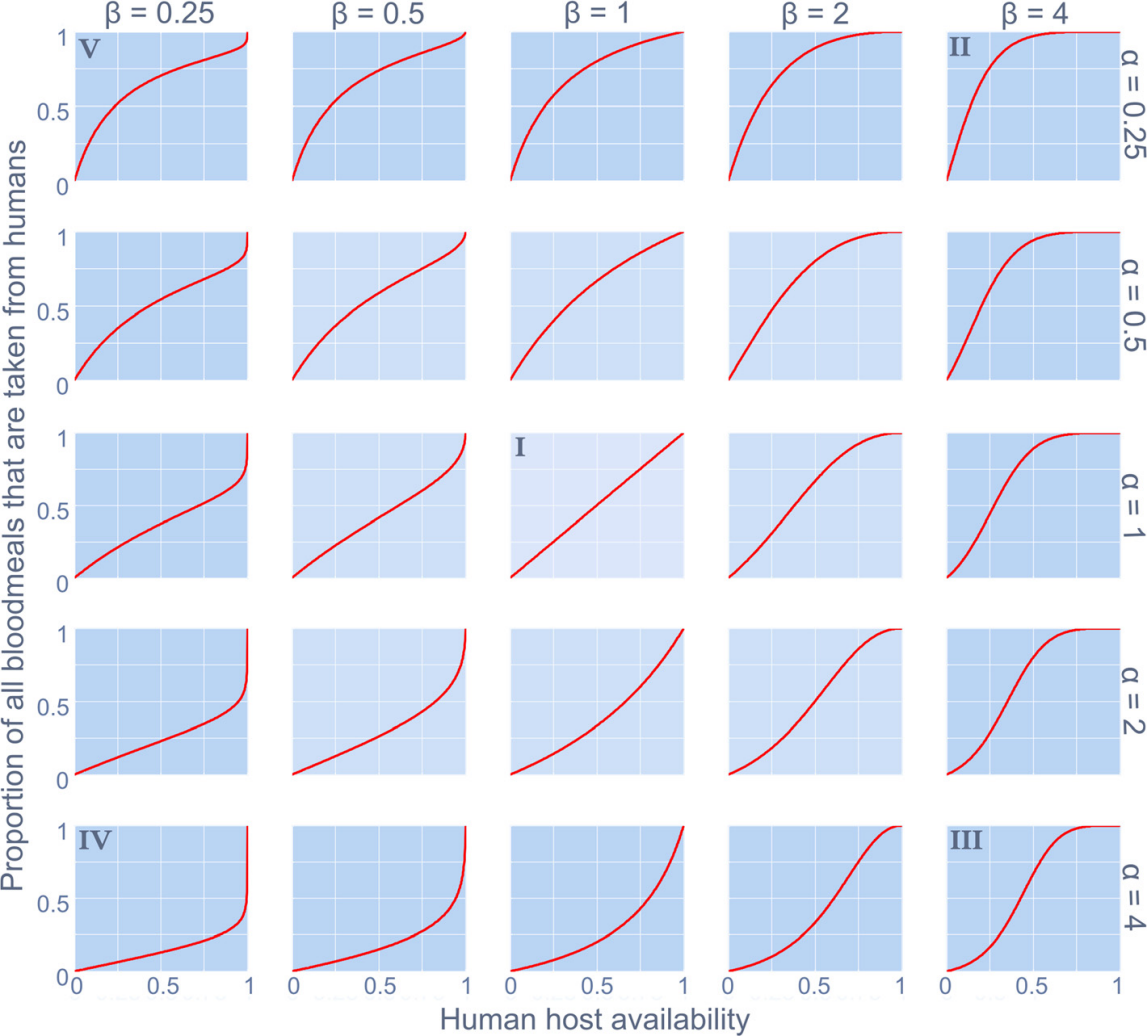
The behavioural response in human blood index of a disease vector to varying levels of human host availability (relative to all potential blood sources). Distinct qualitative forms (denoted ‘I’ to ‘V’) are shaped by parameters α and β as described in Equation 2

The proportion of blood-meals taken from the host species of interest (here, humans) compared to alternative hosts is generally assumed to increase as a direct proportion of increasing relative human availability. A model was sought to relax this key assumption of a century’s worth of vector-borne disease (VBD) models in order to explore the epidemiological impact of non-linear vector biting behavioural responses to host availabilities. While the nested model of Real (1977) (Equation 1) offers a concise and flexible framework for exploring different qualitative responses, there were VBD-specific scenarios that could not be resolved using this existing framework. For example, in the case of a zoonotic VBD which has spilled over into a local human population, a vector with strong zoophilic speciation may only opportunistically bite humans when their preferred host becomes vanishingly rare - this is the entomological/ epidemiological situation reported in Louisiana, USA, where the local kissing bugs (*Triatoma sanguisuga*) were observed to only start biting humans and infecting them with *Trypanosoma cruzi* when the local armadillo population collapsed [22]. This behaviour is not described by a Type I, II or III response. A new, flexible formula was developed to account for a wider range of vector responses to host availability:

2 $$ p = \frac{Q}{Q+\alpha {\left(1-Q\right)}^{\beta }} $$

where, *p* is the proportion of all blood meals that are derived from the species of interest, for humans, this metric has been termed the ‘human blood index’ (HBI) [20]; *Q* is the availability of the host species of interest relative to all potential hosts; α and β are parameters that shape the behavioural response. Figure 1 demonstrates the effects that α and β have on the functional response with this new formulation. In addition to a linear (Type I), convex-up (Type II) and sigmoidal (Type III) response, the new formula also allows for responses that are convex-down (hereon referred to as ‘Type IV’) and atypical sigmoidal (classic s-shape reflected in the y = x, ‘Type V’). The qualitatively distinct feeding behaviours which can be characterised as combinations of intrinsic (genetic) host preferences and vector phenotypic response to local conditions [23] are described in Table 1.

**Table 1:** The qualitatively different behavioural responses (parameterisation and associated vector behaviours) described by the new formula

Response type	Ecological equivalent	Parametric conditions	Vector behaviour
Type I	Analogous to Holling’s Type I	α = 1 β = 1	Indiscriminate; or vector biting that is consistent (proportionate) across relative availabilities of alternative hosts.
Type II	Analogous to Holling’s Type II	α < 1 β ≥ 1	The HBI of an anthropophilic vector saturates whereby even when humans and non-humans have similar availability, almost all blood meals are secured from humans.
Type III	Analogous to Holling’s Type III	α ≥ 1 β > 1	Similar to a Type II response, the HBI saturates, but at low levels of human availability vectors are uninclined to bite them. Corresponding with the analogous Holling’s Type, this could be associated with a learned behaviour with an increased rate of human encounters.
Type IV	Inversion of Holling’s Type II	α > 1 β ≤ 1	A zoophilic vector is uninclined to bite humans until they constitute all but the only available blood source.
Type V	Inversion of Holling’s Type III	α ≤ 1 β < 1	HBI saturates and becomes relatively invariant when humans and non-human hosts are at similar availability. This is analogous to ‘negative prey switching’ whereby the ‘predator’ consumes disproportionately less of the more available ‘prey’ [41]. Eventually, when non-humans become vanishingly rare, the HBI is forced to increase sharply to unity.

The impact of these qualitatively distinct behavioural responses are assessed for different classes of VBDs. Infectious disease agents can be categorised across a spectrum according to their transmission potential to humans relative to non-human species [24]; and a natural, human-centric stratification is to consider the transmission potential to humans of pathogens that are either strict-anthroponotic (where non-human species are incompetent reservoirs), generalist (where humans and non-human species are both competent reservoirs) or strict-zoonotic (where humans are incompetent reservoirs). To exemplify the epidemiological impact of vector biting responses, these three strata are respectively represented by models of falciparum malaria, Chagas disease and Lyme disease.

### Nested ecological-epidemiological models

The transmission dynamics of falciparum malaria, Chagas disease and Lyme disease are all nested within the following general VBD framework:

3 $$ \frac{dS}{dt}=\gamma I+\tau R-{p}_Hm{b}_HSZ $$

4 $$ \frac{dI}{dt}={p}_Hm{b}_HSZ-\left(\gamma \kern0.5em +\kern0.5em \varepsilon +\pi \right)I $$

5 $$ \frac{dR}{dt}=\varepsilon I+\kappa A-\left(\tau +\theta {p}_Hm{b}_HZ\right)R $$

6 $$ \frac{dA}{dt}=\pi I+\theta {p}_Hm{b}_HZR-\kappa A $$

7 $$ \frac{dX}{dt}=\mu V-\left({p}_H{b}_{VH}\left(I+\sigma A\right)+\left(1-{p}_H\right){b}_{VN}\left({I}_N+{\sigma}_N{A}_N\right)\right)X-\mu X $$

8 $$ \frac{dY}{dt}=\left({p}_H{b}_{VH}\left(I+\sigma A\right)+\left(1-{p}_H\right){b}_{VN}\left({I}_N+{\sigma}_N{A}_N\right)\right)X-\left(\zeta +\mu \right)Y $$

9 $$ \frac{dZ}{dt}=\zeta Y-\mu Z $$

10 $$ \frac{d{S}_N}{dt}={\gamma}_N{I}_N+{\tau}_N{R}_N-\left(1-{p}_H\right)m{b}_N{S}_NZ $$

11 $$ \frac{d{I}_N}{dt}=\left(1-{p}_H\right)m{b}_N{S}_NZ-\left({\gamma}_N+{\varepsilon}_N+{\pi}_N\right){I}_N $$

12 $$ \frac{d{R}_N}{dt}={\varepsilon}_N{I}_N+{\kappa}_N{A}_N-\left({\tau}_N+{\theta}_N\left(1-{p}_H\right)m{b}_NZ\right){R}_N $$

13 $$ \frac{d{A}_N}{dt}={\pi}_N{I}_N+{\theta}_N\left(1-{p}_H\right)m{b}_NZ{R}_N-{\kappa}_N{A}_N $$

The epidemiological categories of the vector population and the populations of different host species (subscript *H* refers to humans; *N* refers to non-human hosts) are tracked as proportions. Disease transmission is assumed frequency-dependent to maintain convention with almost all VBD models.

Susceptible hosts (S) become infected (I) following a bite from an infectious vector (Z). Infected hosts can either revert to susceptible at rate γ, or they can benefit from temporary (τ > 0 and/or θ > 0) or permanent (τ = θ = 0) immunity. Alternatively, hosts can become asymptomatically infected (A) directly progressing from symptomatic infection (π > 0) or following on from recovery and subsequent reinfection (θ > 0). Asymptomatic infection may have the same transmission potential to vectors as symptomatic infections (σ = 1) or different transmission potential to vectors (σ ≠ 1), and can either be lifelong (*κ* = 0) or the pathogen can be completely cleared and hosts recover at rate *κ*. Susceptible vectors (X) become *infected* (Y) following a bite from an infectious host, and after the extrinsic incubation period, become *infectious* (Z). Total vectors V = X+Y+Z. Vectors typically outnumber hosts and, following convention, the ratio of vector-to-hosts (all blood-source host species) is denoted *m*. Typically, they also live shorter lives than hosts and so the relatively long extrinsic incubation period is explicitly included, as are vector demographics. Here, a stable vector population is assumed whereby births are set to balance deaths (μ). The impact of biting behaviour is assessed for pathogens with markedly different aetiologies through the following specifications and parameterisations.

### Model specification

Humans are considered the only intermediate host for *Plasmodium falciparum* (c.f. knowlesi malaria for which mixed-host species models now exist [25]). Therefore, transmission terms between vectors and non-human hosts are assumed to equal zero. Following convention of previously published malaria models, host recovery without imparting some level of immunity does not occur; nor does asymptomatic chronicity following initial infection and so γ and π equal zero respectively [26]. The resulting compartmental framework is equivalent to an SIRS model but with possibility of maintained asymptomatic infection status following temporally proximal sequential infections [27].

*Trypanosoma cruzi* is a more generalist pathogen, infecting marsupials, primates, bats, armadillos and rodents, among other species [28]. In highly endemic human communities of Latin America, domestic animals are the key infection source and dogs are the primary parasite reservoir [29]. Infection dynamics are therefore tracked between dogs, the kissing bug vector and humans. (However, Equations 3–13 can be extended to greater numbers of blood-hosts and this will constitute important future work). Similarly for dogs and humans, infection is not cleared and acute infection invariably leads to chronic, asymptomatic infection. Hence, ε, γ, τ, θ and κ equal zero and the transitions are described by an SIA model. A key difference between these two blood-hosts is that while asymptomatically infected dogs can continue to transmit the parasite to vectors (σ_N_ > 0), chronically infected humans do not constitute parasite reservoirs [30].

Humans are dead-end hosts of Lyme disease. Therefore, human host transmission to the vector (*b*_*VH*_) equals zero. No temporary or lasting immunity has been documented for humans and so dynamics are described by an SIS model; whereas infection is generally chronic and symptomless in amplification host species (e.g. white-footed field mice, deer) and described by an SIA model [31]. These substructures can be achieved as with the other disease examples by setting the redundant rates to zero. Rates of change between the remaining epidemiological categories for all infection models are described in full in Table 2.

**Table 2:** Parameterisation for vector-borne disease models

	Definition	*Plasmodium falciparum*	*Trypanosoma cruzi*	*Borrelia burgdorferi*
*b*_*i*_	Transmission coefficient (vectors→hosts) = bite rate x transmission probability	0.1 = 1/3 × 0.3 (humans) [42]; 0 (non-humans)	2 × 10^-5^ = ¼ × 8 × 10^-5^ (humans) [43, 44]; 2.5 × 10^-4^ = ¼ × 0.001 (non-humans) [45]	$0.003=\frac{1}{365}\kern0.5em \times \kern0.5em 1.0\ \left(\mathrm{humans}\ \mathrm{and}\ \mathrm{n}\mathrm{o}\mathrm{n}\hbox{-} \mathrm{humans}\right)$ [46]
*b*_*Vi*_	Transmission coefficient (hosts→vectors) = bite rate x transmission probability	$$ 0.007=\raisebox{1ex}{$1$}\!\left/ \!\raisebox{-1ex}{$3$}\right.\kern0.5em \times \kern0.5em 0.02\kern0.5em \left(\mathrm{humans}\right) $$ [47]; $$ 0\kern0.5em \left(\mathrm{n}\mathrm{o}\mathrm{n}{\textstyle \hbox{-}}\mathrm{humans}\right) $$	0.015 = ½ × 0.03 (humans); 0.25 = ½ × 0.49 (non-humans) [48]	$$ 0\ \left(\mathrm{humans}\right); $$ $$ 0.003\kern0.5em =\kern0.5em \frac{1}{365}\kern0.5em \times \kern0.5em 1.0\ \left(\mathrm{n}\mathrm{o}\mathrm{n}\hbox{-} \mathrm{humans}\right) $$ [46]
*γ*	Recovery rate (no immunity)	0 (humans and non-humans)	0 (humans and non-humans)	1/28 (humans)^a^; 0 (non-humans) [31]
ε	Clearance rate of symptomatic infection	1/200 (humans) [49]; 0 (non-humans)	0 (humans and non-humans)	0 (humans and non-humans)
κ	Clearance rate of asymptomatic infection	1/200 (humans) [49]; 0 (non-humans)	0 (humans and non-humans)	0 (humans and non-humans)
π	Asymptomatic primary infection rate	0 (humans and non-humans)	1/40 (humans and non-humans) [50, 51]	0 (humans); 1/28 (non-humans) [31]
θ	Asymptomatic secondary infection rate	0.5 (assumed for humans); 0 (non-humans)	0 (humans and non-humans)	0 (humans and non-humans)
τ	Full susceptibility reversion rate	1/1000 (humans) [52]; 0 (non-humans)	0 (humans and non-humans)	0 (humans and non-humans)
μ	Birth (or maturation) and death rate of vectors (i.e. stable population)	1/10 [53]	1/365 [54]	1/365 [55]
σ	Adjustment factor for asymptomatic transmissibility to vector	0.25 (humans) [56]; 0 (non-humans)	≈0 humans [30]; ≈1 non-humans [57]^b^	0 (humans); ≈1 (non-humans)
ζ	Rate of parasite development within vector	1/10 [58]	1/10 [59]	1/365 [60]^c^

^a^Classically, Lyme disease infection dynamics are of an SIS form whereby the pathogen is assumed to be cleared by the host’s immune system. However, Nadelman & Wormser [31] review several studies demonstrating that an SIA form is more appropriate for non-human hosts

^b^A longitudinal study of domestic dogs (a principal Chagas disease reservoir) demonstrated persistent infectiousness but it was unclear whether this was a result of repeat infections

^c^Parasite development is assumed to correspond with the developmental delays between life stages of the tick (whereby the tick will take its blood-meal from a different host species)

## Results

**Fig. 2:**
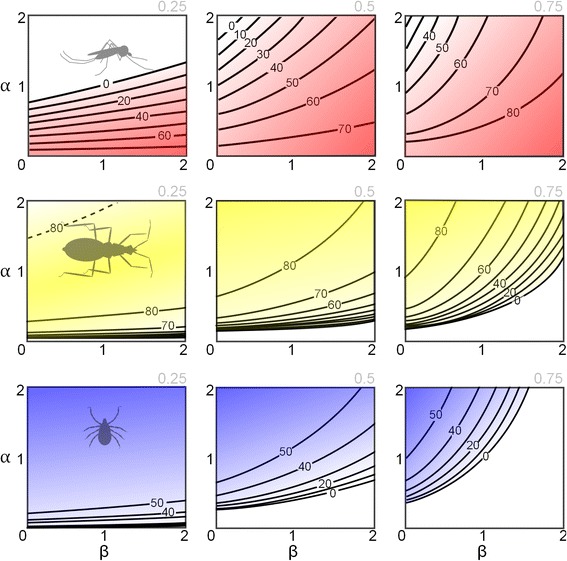
Disease control efficacy is contingent on the behavioural response of biting vectors to the availability of alternative blood-hosts. The parameters α and β determine the shape of the behavioural response as described in Equation 2. The human proportion of all blood-hosts is indicated in the top-right of each plot. For *Plasmodium falciparum* (top row), the region above the contours corresponds with controlled transmission, but for *Trypanosoma cruzi* (middle row) and *Borrelia burgdorferi* (bottom row), the regions below the contours correspond with controlled transmission. A special case is shown in the left plot for *T. cruzi* whereby the high α/ low β region (above broken line) delimits a second parameter space for controlled transmission (see text). The contour labels correspond with the percentage reduction in bite rate required to achieve control. (These models are all deterministic and so a 90 % reduction in the acute infections relative to the maximum level in the absence of control is used to infer controlled transmission)

Control strategies focusing on reducing vector biting rates were simulated for models representing each of the three pathogen life-cycle strata. Specifically, the impact on control of different behavioural responses in vector biting behaviour was assessed separately for falciparum malaria, Chagas disease and Lyme disease (Fig. 2). Due to their long duration, rates of chronic infection with vector-borne diseases are slow to change following control and so results are presented in terms of the impact of vector behavioural response on rates of acute (symptomatic) infections which better characterise disease incidence.

Intuitively, acute malaria infection was easier to control when vectors had strong zoophilic preference across a wide range of host availabilities (high α, low β, i.e. Type IV) and most resilient to control when vectors were highly anthropophilic across a wide range of host availabilities (low α, high β i.e. Type II). Also expected for this parasite that requires passage through humans for its propagation, was the result that when humans constituted a larger proportion of the total blood-host availability, infection was more difficult to control (from left to right on top row of Fig. 2).

Acute human infection with both Lyme and Chagas disease had qualitatively similar relationships with the vectors’ behavioural response. Control was easiest when vectors were highly anthropophilic (low α, high β, i.e. Type II), and hardest when vectors were zoophilic (high α, low β i.e. Type IV). While this was an expected result for Lyme disease (whereby humans are dead-end hosts and thereby detract from the pathogen transmission cycle), the qualitatively similar result for Chagas was less intuitive and arose from the disproportionately high contribution of non-human hosts to the force of infection (dogs not only remain infectious for much longer than humans but are substantially more infectious to the vectors, Table 2). Host preference that was weighted against humans (high α, low β, i.e. Type IV) was most resilient to human infection control for all Lyme and Chagas disease scenarios, with one exception. For a generalist pathogen (*T. cruzi*), there are some circumstances when a more intermediate biting behaviour (less definitively anthropophilic/zoophilic) can give rise to a human infection that is less amenable to control (left subplot, middle row of Fig. 2). Here, a Type II response (low α, high β) reduces the proportion of bites on the more competent host (dogs), in turn reducing the prevalence of disease and subsequent force of infection on humans. However, when humans only constitute a minority of available blood-hosts, the exaggerated proportion of bites on dogs under a Type IV response (high α, low β) depletes the opportunities for pathogen spread to humans more than the reduced prevalence of disease that would result for a less zoophilic vector. Therefore, both extremes of vector biting behaviour are more conducive to control.

## Discussion

Models of diseases spread by haematophagous arthropods are increasingly popular tools for understanding the spread of vector-borne diseases and strategizing their control. Persisting among even the most complex of contemporary models is the widespread assumption that vector bites are distributed in a directly proportionate manner on alternative hosts according to their relative availability, sometimes adjusted according to a constant intrinsic host preference as described by Bailey [32]. Critically, a linear (Type I) functional response is not only atypical for arthropods, it is without precedent [33].

Unfortunately, examples of studies that account for non-linear effects of human host availability on vector biting behaviour are scant. While parallel advances have been made in the context of predator-prey [34] and host-parasitoid systems [35], these have not translated to corresponding developments in VBD understanding. Antonovics et al. [36] used a general Type II model to simulate VBD transmission and described how a vector that is highly restricted in its movement could well have its bite rate limited by low host density (invalidating the general assumption of frequency-dependence); and, frequency-dependence and density-dependence are well known to generate markedly different transmission dynamics [37]. This concept has been recently built upon by Kershenbaum et al. [38] who showed in a one-vector two-host (one competent, one incompetent) model that parameter spaces exist whereby a reduction in competent host availability risks exacerbating disease prevalence through attenuated competition for limited vector feeding sites. The current study does not account for this potential transmission bottleneck; and a unified approach to account for not only the proportional distribution of bites across different alternative host availabilities but also a vector biting rate that can be influenced by host densities constitutes an important future endeavour.

A further aspect of the current work that justifies future development is the exploration of how different behavioural responses might impact pathogen transmission dilution and amplification [39]. Miller & Huppert [40] also used a Type II formulation to describe vector bites split between multiple host species and showed disease transmission is intensified (or ‘amplified’) when more host species are included in a system if the vector prefers the host with the highest transmission ability; otherwise, the addition of more host species dilutes transmission [40]. Although the current study is restricted to a vector shared between only two alternative hosts, the methods described are easily adaptable to more hosts for the analysis of species diversity and pathogen persistence.

## Conclusions

This study makes the following contributions: it provides a new, two-parameter function that can be used to understand functional response ecology and that extends the qualitative Types achievable with earlier (three-parameter) models of Holling and Real; it introduces a general framework into which this vector behaviour can be incorporated to explore its consequences on infectious diseases with diverse epidemiology; it highlights an aspect of vector behaviour that is almost completely neglected; and demonstrates how this response of the vector to alternative host availabilities can drastically alter efforts to mitigate transmission. Data derived from laboratory or semi-field conditions (where the relative availabilities of alternative blood hosts can more easily be manipulated) are a high research priority in the field of vector-borne disease. Diverse aetiologies and idiosyncratic epidemiology severely limit opportunities for scientific discoveries that can potentially impact the whole gamut of vector-borne diseases. The ecology of this behaviour in biting disease vectors potentially offers one of the last largely unexplored avenues of generally applicable vector-borne disease research.

## Abbreviations

HBI: Human blood index
SIA: Susceptible-infected-asymptomatic
SIRS: Susceptible-infected-recovered-susceptible
SIS: Susceptible-infected-susceptible
VBD: Vector-borne disease

## References

[1] Holling CS (1959). The components of predation as revealed by a study of small mammal predation of the European pine sawfly. Can Entomol.

[2] Holling CS (1959). Some characteristics of simple types of predation and parasitism. Can Entomol.

[3] Holling CS (1965). The functional response of predators to prey density and its role in mimicry and population regulation. Mem Entomol Soc Can.

[4] Solomon ME (1949). The natural control of animal populations. J Anim Ecol.

[5] Lafferty KD, DeLeo G, Briggs CJ, Dobson AP, Gross T, Kuris AM (2015). A general consumer-resource population model. Science.

[6] Real LA (1977). The kinetics of functional response. Am Nat.

[7] McCallum H (2000). Preditor-prey, host-parasitoid and plant-herbivore models. Population parameters: estimation for ecological models.

[8] Lehane MJ (2005). The Biology of Blood-Sucking in Insects.

[9] Chaves LF, Harrington LC, Keogh CL, Nguyen AM, Kitron UD (2010). Blood feeding patterns of mosquitoes: random or structured?. Front Zool.

[10] Rabinovich JE, Kitron UD, Obed Y, Yoshioka M, Gottdenker N, Chaves LF (2011). Ecological patterns of blood-feeding by kissing-bugs (Hemiptera: Reduviidae: Triatominae). Mem I Oswaldo Cruz.

[11] Pates HV, Takken W, Stuke K, Curtis CF (2001). Differential behaviour of *Anopheles gambiae* sensu stricto (Diptera: Culicidae) to human and cow odours in the laboratory. B Entomol Res.

[12] White GB, Magayuka SA, Boreham PFL (1972). Comparative studies on sibling species of the *Anopheles gambiae* Giles complex (Dipt., Culicidae): bionomics and vectorial activity of species A and species B at Segera, Tanzania. B Entomol Res.

[13] Lyimo IN, Keegan SP, Ranford-Cartwright LC, Ferguson HM (2012). The impact of uniform and mixed species blood meals on the fitness of the mosquito vector *Anopheles gambiae* s.s: Does a specialist pay for diversifying its host species diet?. J Evolution Biol.

[14] Abrams PA, Matsuda H (2003). Population dynamical consequences of reduced predator switching at low total prey densities. Popul Ecol.

[15] Rueffler C, VanDooren TJM, Metz JAJ (2004). Adaptive walks on changing landscapes: Levins’ approach extended. Theor Popul Biol.

[16] Önder Ö, Shao W, Lam H, Brisson D (2014). Tracking the sources of blood meals of parasitic arthropods using shotgun proteomics and unidentified tandem mass spectral libraries. Nat Protocols.

[17] Saul A (2003). Zooprophylaxis or zoopotentiation: the outcome of introducing animals on vector transmission is highly dependent on the mosquito mortality while searching. Malaria J.

[18] Iwashita H, Dida GO, Sonye GO, Sunahara T, Futami K, Njenga SM, Chaves LF, Minakawa N (2014). Push by a net, pull by a cow: can zooprophylaxis enhance the impact of insecticide treated bed nets on malaria control?. Parasit Vectors.

[19] Yakob L (2016). Endectocide-treated cattle for malaria control: A coupled entomological-epidemiological model. Parasite Epidemiol Control.

[20] Garrett-Jones C (1964). The human blood index of malaria vectors in relation to epidemiological assessment. B World Health Organ.

[21] Killeen GF, Chitnis N, Moore SJ, Okumu FO (2011). Target product profile choices for intra-domiciliary malaria vector control pesticide products: repel or kill?. Malaria J.

[22] Dorn PL, Perniciaro L, Yabsley MJ, Roellig DM, Balsamo G, Diaz J, Wesson D (2007). Autochthonous transmission of *Trypanosoma cruzi*, Louisiana. Emerg Infect Dis.

[23] Lefèvre T, Gouagna L-C, Dabiré KR, Elguero E, Fontenille D, Renaud F (2009). Beyond Nature and Nurture: Phenotypic plasticity in blood-feeding behavior of *Anopheles gambiae *s.s. when humans are not readily accessible. Am J Trop Med Hyg.

[24] Wolfe ND, Dunavan CP, Diamond J (2007). Origins of major human infectious diseases. Nature.

[25] Yakob L, Bonsall MB, Yan G (2010). Modelling knowlesi malaria transmission in humans: vector preference and host competence. Malaria J.

[26] Mandal S, Sarkar RR, Sinha S (2011). Mathematical models of malaria - a review. Malaria J.

[27] Artzy-Randrup Y, Dobson AP, Pascual M (2015). Synergistic and antagonistic interactions between bednets and vaccines in the control of malaria. Proc Natl Acad Sci U S A.

[28] Noireau F, Diosque P, Jansen AM (2009). *Trypanosoma cruzi*: adaptation to its vectors and its hosts. Vet Res.

[29] Gurtler RE, Cecere MC, Lauricella MA, Cardinal MV, Kitron U, Cohen JE (2007). Domestic dogs and cats as sources of *Trypanosoma cruzi* infection in rural northwestern Argentina. Parasitol.

[30] Añez N, Martens ML, Romero M, Crisante G (2011). *Trypanosoma cruzi* primary infection prevents severe re-infection in mice. Bol Mal Salud Ambiental.

[31] Nadelman RB, Wormser GP (2007). Reinfection in patients with Lyme disease. Clin Infect Dis.

[32] Bailey NTJ (1982). The Biomathematics of Malaria.

[33] Jeschke JM, Kopp M, Tollrian R (2004). Consumer-food systems: why type I functional responses are exclusive to filter feeders. Biol Rev.

[34] Oaten A, Murdoch WW (1975). Switching, functional response, and stability in predator-prey systems. Am Nat.

[35] Hassell MP (1978). The dynamics of arthropod predator-prey systems.

[36] Antonovics J, Iwasa Y, Hassell MP (1995). A generalized model of parasitoid, venereal, and vector-based transmission processes. Am Nat.

[37] Getz WM, Pickering J (1983). Epidemic models: thresholds and population regulation. Am Nat.

[38] Kershenbaum A, Stone L, Ostfeld RS, Blaustein L (2012). Modelling transmission of vector-borne pathogens shows complex dynamics when vector feeding sites are limited. PLoS One.

[39] Randolph SE, Dobson ADM (2012). Pangloss revisited: a critique of the dilution effect and the biodiversity-buffers-disease paradigm. Parasitol.

[40] Miller E, Huppert A (2013). The effects of host diversity on vector-borne disease: The conditions under which diversity will amplify or dilute the disease risk. PLoS One.

[41] Abrams PA, Matsuda H, Harada Y (1993). Evolutionarily unstable fitness maxima and stable fitness minima of continuous traits. Evol Ecol.

[42] Rickman L, Jones TR, Long GW, Paparello S, Schneider I, Paul CF (1990). *Plasmodium falciparum*-infected *Anopheles stephensi* inconsistently transmit malaria to humans. Am J Trop Med Hyg.

[43] Coffield DJ, Spagnuolo AM, Shillor M, Mema E, Pell B, Pruzinsky A, Zetye A (2013). A Model for Chagas disease with oral and congenital transmission. PLoS One.

[44] Catalá S (1991). The biting rate of *Triatoma infestans* in Argentina. Med Vet Entemol.

[45] Cohen JE, Gürtler RE (2001). Modeling household transmission of American trypanosomiasis. Science.

[46] Piesman J, Mather TN, Sinsky RJ, Spielman A (1987). Duration of tick attachment and *Borrelia burgdorferi* transmission. J Clin Microbiol.

[47] Bonnet S, Gouagna LC, Paul RE, Safeukui I, Meunier JY, Boudin C (2003). Estimation of malaria transmission from humans to mosquitoes in two neighbouring villages in south Cameroon: evaluation and comparison of several indices. T Roy Soc Trop Med H.

[48] Gurtler RE, Cecere MC, Castanera MB, Canale D, Lauricella MA, Chuit R, Segura EL (1996). Probability of infection with *Trypanosoma cruzi* of the vector *Triatoma infestans* fed on infected humans and dogs in northwest Argentina. Am J Trop Med Hyg.

[49] Falk N, Maire N, Sama W, Owusu-Agyei S, Smith T, Beck H, Felger I (2006). Comparison of PCR-RFLP and Genescan-based genotyping for analyzing infection dynamics of *Plasmodium falciparum*. Am J Trop Med Hyg.

[50] Gomes ML, Toledo MJO, Nakamura CV, Bittencourt NLR, Chiari E, Araújo SM (2003). *Trypanosoma cruzi*: genetic group with peculiar biochemical and biological behavior. Mem I Oswaldo Cruz.

[51] Teixeira ARL, Hecht MM, Guimaro MC, Sousa AO, Nitz N (2011). Pathogenesis of Chagas' disease: Parasite persistence and autoimmunity. Clin Microbiol Rev.

[52] Doolan DL, Dobaño C, Baird JK (2009). Acquired immunity to malaria. Clin Microbiol Rev.

[53] Ermert V, Fink A, Jones A, Morse A (2011). Development of a new version of the Liverpool Malaria Model. I. Refining the parameter settings and mathematical formulation of basic processes based on a literature review. Malaria J.

[54] Chaves LF, Hernandez M-J, Revilla TA, Rodríguez DJ, Rabinovich JE (2004). Mortality profiles of *Rhodnius prolixus* (Heteroptera: Reduviidae), vector of Chagas disease. Acta Trop.

[55] Apanaskevich DA, Oliver JH, Sonenshine DE, Roe RM (2014). Life cycles and natural history of ticks. Biology of Ticks.

[56] Okell LC, Bousema T, Griffin JT, Ouedraogo AL, Ghani AC, Drakeley CJ (2012). Factors determining the occurrence of submicroscopic malaria infections and their relevance for control. Nat Commun.

[57] Gürtler RE, Lauricelia M, Solakz ND, Bujas MA, Wisnivesky-Colli C (1986). Dynamics of transmission of *Trypanosoma cruzi* in a rural area of Argentina. I The dog reservoir: an epidemiological profile. Rev I Med Trop São Paulo.

[58] Blanford JI, Blanford S, Crane RG, Mann ME, Paaijmans KP, Schreiber KV, Thomas MB. Implications of temperature variation for malaria parasite development across Africa. Sci Rep. 2013;3:1300. 10.1038/srep01300 PMC3575117 23419595

[59] Paniker CJ (1988). Medical Parasitology.

[60] Kurtenbach K, Hanincová K, Tsao JI, Margos G, Fish D, Ogden NH (2006). Fundamental processes in the evolutionary ecology of Lyme borreliosis. Nat Rev Micro.

